# A Robust Training Method for Pathological Cellular Detector *via* Spatial Loss Calibration

**DOI:** 10.3389/fmed.2021.767625

**Published:** 2021-12-14

**Authors:** Hansheng Li, Yuxin Kang, Wentao Yang, Zhuoyue Wu, Xiaoshuang Shi, Feihong Liu, Jianye Liu, Lingyu Hu, Qian Ma, Lei Cui, Jun Feng, Lin Yang

**Affiliations:** ^1^School of Information Science and Technology, Northwest University, Xi'an, China; ^2^Fudan University Shanghai Cancer Center, Shanghai, China; ^3^Department of Computer Science and Engineering, University of Electronic Science and Technology of China, Chengdu, China; ^4^AstraZeneca, Shanghai, China

**Keywords:** cellular detection, spatial loss calibration, sparsely annotated pathological datasets, convolutional neural network, object detection network

## Abstract

Computer-aided diagnosis of pathological images usually requires detecting and examining all positive cells for accurate diagnosis. However, cellular datasets tend to be sparsely annotated due to the challenge of annotating all the cells. However, training detectors on sparse annotations may be misled by miscalculated losses, limiting the detection performance. Thus, efficient and reliable methods for training cellular detectors on sparse annotations are in higher demand than ever. In this study, we propose a training method that utilizes regression boxes' spatial information to conduct loss calibration to reduce the miscalculated loss. Extensive experimental results show that our method can significantly boost detectors' performance trained on datasets with varying degrees of sparse annotations. Even if 90% of the annotations are missing, the performance of our method is barely affected. Furthermore, we find that the middle layers of the detector are closely related to the generalization performance. More generally, this study could elucidate the link between layers and generalization performance, provide enlightenment for future research, such as designing and applying constraint rules to specific layers according to gradient analysis to achieve “scalpel-level” model training.

## 1. Introduction

Locating and counting cells in the pathological whole slide images (WSIs) is a direct way to find effective and important biomarkers, which is an essential and fundamental task of pathological image analysis (1–3). For instance, the spatial arrangement of tumor cells has been proved to be related to cancer grades (4, 5). Therefore, the qualitative and quantitative analysis of different types of tumors at cellular-level detection can help us better understand tumors and also explore various options for cancer treatment (6, 7).

**Figure 1:**
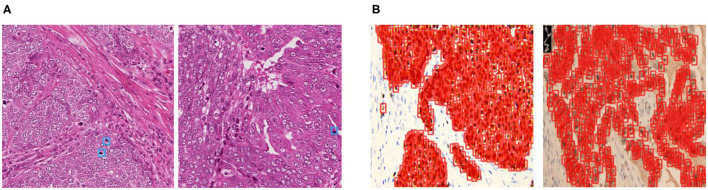
The examples of annotations in the two kinds of datasets. **(A)** Cells of embedded distribution, which are sampled from the MITOS-ATYPIA-14 dataset. Obviously, mitoses that need to be annotated are often hidden among hundreds of other cells, tough to categorize and locate. **(B)** Cells of dense distribution in our Ki-67 dataset, usually more than hundreds of cells are required to be annotated in a small patch sampled from the whole slide image (WSI), which is an expensive and laborious task.

Recently, object detection frameworks of Convolutional Neural Networks (obj-CNNs) have been proved powerful for locating instances in medical images [e.g., in CT images (8) and colonoscopy images (9)]. The big empirical success of obj-CNNs depends on the availability of a large corpus of fully annotated instances in training images (10). However, different from images of other modalities, we find two kinds of distributions of cells in pathological images, namely embedded and dense distribution, making full annotations of cellular-level instances difficult to be guaranteed (refer to Figure 1). Specifically, the embedded distribution means that positive cells are hidden among hundreds of other cells, which are challenging for pathologists to categorize, locate, and then annotate. As for the dense distribution, a small patch sampled from the WSIs may contain hundreds of positive cells, making the annotation task expensive and laborious. Therefore, sparsely annotated datasets (SADs) are common in the field of the detection of cells.

In fact, when the training dataset contains a certain amount of sparse cellular annotations, the overfitting issue tends to easily occur, naturally leading to poor performance in generalization (11). In this study, we show the fundamental problems that decrease the generalization performance of the detector trained on SADs. First, deviation-loss, that is, numerous unannotated positive cells are mistaken for negative ones in the SADs, resulting in a serious miscalculated loss during training. Second, the deviation-loss dominates the early training process, and then drives the detector to learn only the features of the annotated cells, which yields the overfitting issue (Experimental testify can be seen in Appendix A1).

**Figure 2:**
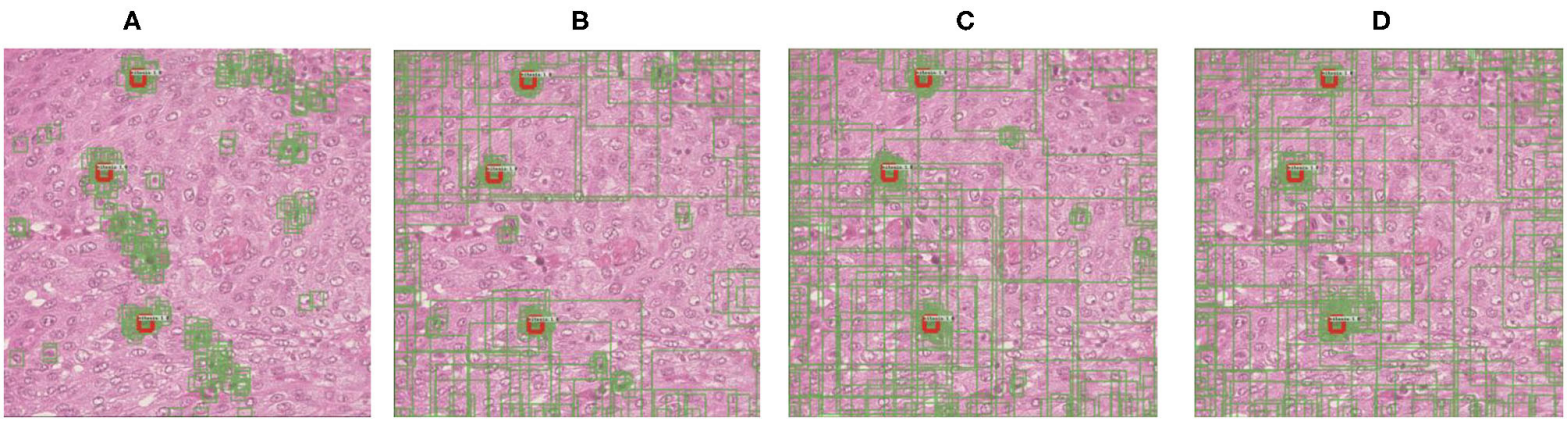
The change of regression boxes' density during training on the MITOS-ATYPIA-14 dataset. Among them, the regression box is in green and manually annotated as red. This typical example shows that as the training process progresses, regression boxes increasingly surround the positive cells. **(A)** Regression boxes in 1 k training steps. **(B)** Regression boxes in 4 k training steps. **(C)** Regression boxes in 7 k training steps. **(D)** Regression boxes in 10 k training steps.

In this study, we point out that alleviating the deviation-loss during the training process can guide the detector to continuously learn the features of positive cells rather than only the annotated ones, and the SADs overfitting problem can be solved. In order to achieve that goal, the first cornerstone is how to identify those positive cells from negative ones when annotations are missing. We observe the more and more significant difference in densities between the predictions of the positive and negative cells during training (refer to Figure 2). Based on this observation, we propose a SADs training method named Boxes Density Energy (BDE), which utilizes densities' information to reduce the deviation-loss. Specifically, the more predictions for a cell, the more likely the cell is to be positive, and these predictions deserve smaller losses. In this way, deviation-loss disappears, and meanwhile, the overfitting problem is solved naturally.

We have conducted experiments on two datasets, namely the MITOS-ATYPIA-14 dataset (embedded distribution)^1^ and the Ki-67 dataset (dense distribution), which can be seen in Figure 1. Sufficient experimental results prove that our training method can significantly boost the performance of SADs. More importantly, we explore the gradient in the network and find that BDE brings a significant improvement on the middle layers (20–60 layers, 80 layers in total) of the network, indicating that the network's generalization performance seems to be closely related to the middle layers of the network. This may change the current training paradigm, such as applying constraint rules to specific layers according to gradient analysis to achieve the “scalpel-level" model training.

The organization of the study is as follows. The review of obj-CNNs and recent literature on SADs training methods is given in Section 2. Section 3 describes the proposed method in detail, and experimental results are presented in Section 4. Finally, we analyze the gradient of the trained network and conclude in Sections 5, 6, respectively.

A preliminary version of this study has been published in a conference study (12), which is only evaluated on the MITOS-ATYPIA-14 dataset. In this study, we have made significant extensions to generalize our methods on the Ki-67 dataset, aiming to provide a strong and comprehensive theory for relevant research. To be specific,

- We explore that some specific layers of CNN are strongly related to generalization performance, may provide theoretical guidance for future related research, e.g., one can improve the generalization of the network through more constraints on middle layers when training the network.
- In this study, we define the networks' training problems on SADs, from deviation-loss to the overfitting issue.
- This study formulated two cells' distribution in pathological images, namely embedded and dense distribution which may easily lead to SADs, and BDE can solve the SADs training problem on both embedded and dense distributions.

## 2. Related Study

### 2.1. Object Detection Networks

#### 2.1.1. The Framework

**Figure 3:**
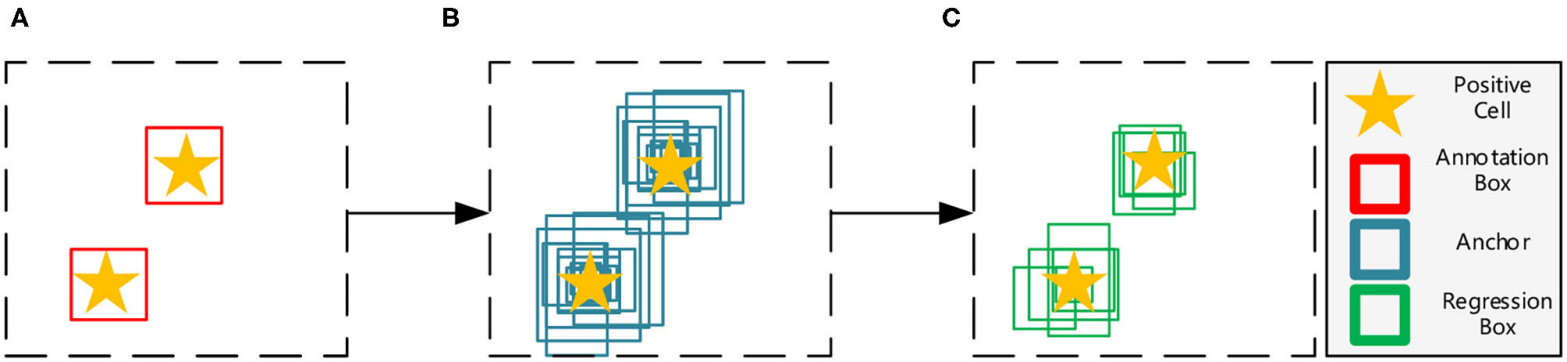
Illustration of the anchor based two-stage framework. **(A)** The input image with manual annotations. **(B)** The first stage refines the initial thousands of anchors. **(C)** The second stage refines previous results and obtains hundreds of regression boxes.

Object detection networks can be divided into two major categories, anchor-free and anchor-based frameworks. Among them, anchor-free frameworks (13, 14) are essentially making dense predictions, receiving higher recall rates but lower accuracy results (15), which do not meet the requirement of precisely pathological image analysis. On the other hand, anchor-based frameworks are more suitable for our tasks, and can be generally divided into one-stage methods (16, 17) and two-stage methods (18, 19). Both of them first tile a large number of preset anchors on the image, then predict the category and refine the coordinates of these anchors by one or several times, finally output these refined anchors as detection results. Because two-stage frameworks refine anchors several times more than one-stage frameworks (as shown in Figure 3), the former has greater accuracy. Hence, we choose the two-stage Feature Pyramid Network (FPN) (19) as the baseline in this paper.

#### 2.1.2. The Loss Function and Deviation Loss

In order to locate and recognize positive cells in the image, the object detection network has two parallel output layers to generate regression boxes (*b*) with probability distribution (*p*). The original loss (*L*) consists of the classification loss *L*_cls_ and bounding-box regression loss *L*_loc_:

(1) $$\begin{array}{r} L\left(p,u,b,v\right)=L_{\text{cls}}\left(p,u\right)+L_{\text{loc}}\left(b,v\right),, \end{array}$$

(2) $$\begin{array}{r} L_{cls}\left(p,u\right)=\underset{k}{\sum }-\left[u_{k}\cdot \log\left(p_{k}\right)\right], \end{array}$$

(3) $$\begin{array}{r} L_{\text{loc}}\left(b,v\right)=\underset{k}{\sum }{smooth}_{L_{1}}\left(b_{k}-v_{k}\right), \end{array}$$

(4) $$smooth_{L_{1}}(x)=\{\begin{array}{ll} 0.5x^{2} & if|x|<1\\ |x|-0.5 & otherwise. \end{array}$$

In Equation (2), *u*_*k*_ represents a one-hot label for a regression box indexed by *k*. When *k*-box's Intersection Over Union (IoU) with any instance annotation higher than a threshold, is assigned with a positive one-hot label (*u*_*k*_ ≠ 0), otherwise a negative (*u*_*k*_ = 0). In Equation 3, *v* indicates the annotated bounding-boxes.

The loss function can accurately measure the margins between *p* and *u*, *b*, and *v* on the fully annotated dataset. However, on the sparsely annotated cellular dataset, all unannotated positive cells are mistaken for negative, and *u* and *v* are translated into “untrustworthy” ground-truths. Thus, *L*_cls_ and *L*_loc_ may deviate seriously from the correct value, which we name deviation-loss. As a result, the deviation-loss confuses the training of networks, leading to limited performance.

### 2.2. Sparsely Annotated Datasets Training Methods

#### 2.2.1. Pseudo-Annotation Based Methods

In order to solve the SADs training problem, pseudo-annotation based methods have been proposed and achieved success on natural images (20, 21). They first train the detector using available instance-level annotations, then generate pseudo-annotations, and merge them with the original annotations to iteratively update the detector. For example, Niitani et al. (22) trained the detector to generate annotations using the Open Images Dataset V4 (OID). They then sampled the pseudo-annotations using assumptions such as “cars should contain tires.” However, such a priori assumption in the field of cell detection is unknown. Other methods based on pseudo-annotations still need a certain number of fully annotated datasets, like Yan et al. (23) and Inoue et. al. (24) employ a subset of fully annotated datasets to obtain a pre-trained detector, generating pseudo-annotations for the next training.

Obviously, such an iterative process brings uncontrollability into the training process, e.g., a bad pseudo-annotation generator may significantly influence the final results. In addition, there is not much consensus on how to utilize the pseudo-annotations until now, especially for object detection (22), e.g., determining the optimal number of iterations is tricky, therefore, it is urgent to solve the SADs training problem in a non-iterative way. Besides, considering that such methods are relatively difficult to replicate, with respect to, empirical and tricky parameter selection or special requirements of the forms of datasets, this study does not include such methods in the comparative experiment.

### 2.3. Loss-Calibration Based Methods

Compared with pseudo-annotation based methods, the loss-calibration methods for solving noise labels are more relevant to our study. The meaning of noise labels is wrong labels or missing labels (25, 26). These methods aim to reduce noise labels by establishing loss functions that are more noise-tolerant. For example, Müller et al. (27) softens the labels by adding a uniform distribution. Wang et al. (28) assumes that the network will become more and more reliable as the training continues and proposes reducing the loss gradually to reduce the influence of noise labels. However, these loss calibration methods also inevitably reduce the core contributions of correct labels for the training of the network. On the contrary, our BDE utilizes the regression boxes' density to encourage correct predictions and give relatively more significant losses to wrong predictions, whether the label is missing or not.

It is worth noting that in view of the class imbalance problem they, the have put forward many loss weighting schemes (17, 29). However, these methods may cause relatively large losses to correct predictions lacking corresponding annotations, which makes them ineffective on SADs.

## 3. Boxes Density Energy

**Figure 4:**
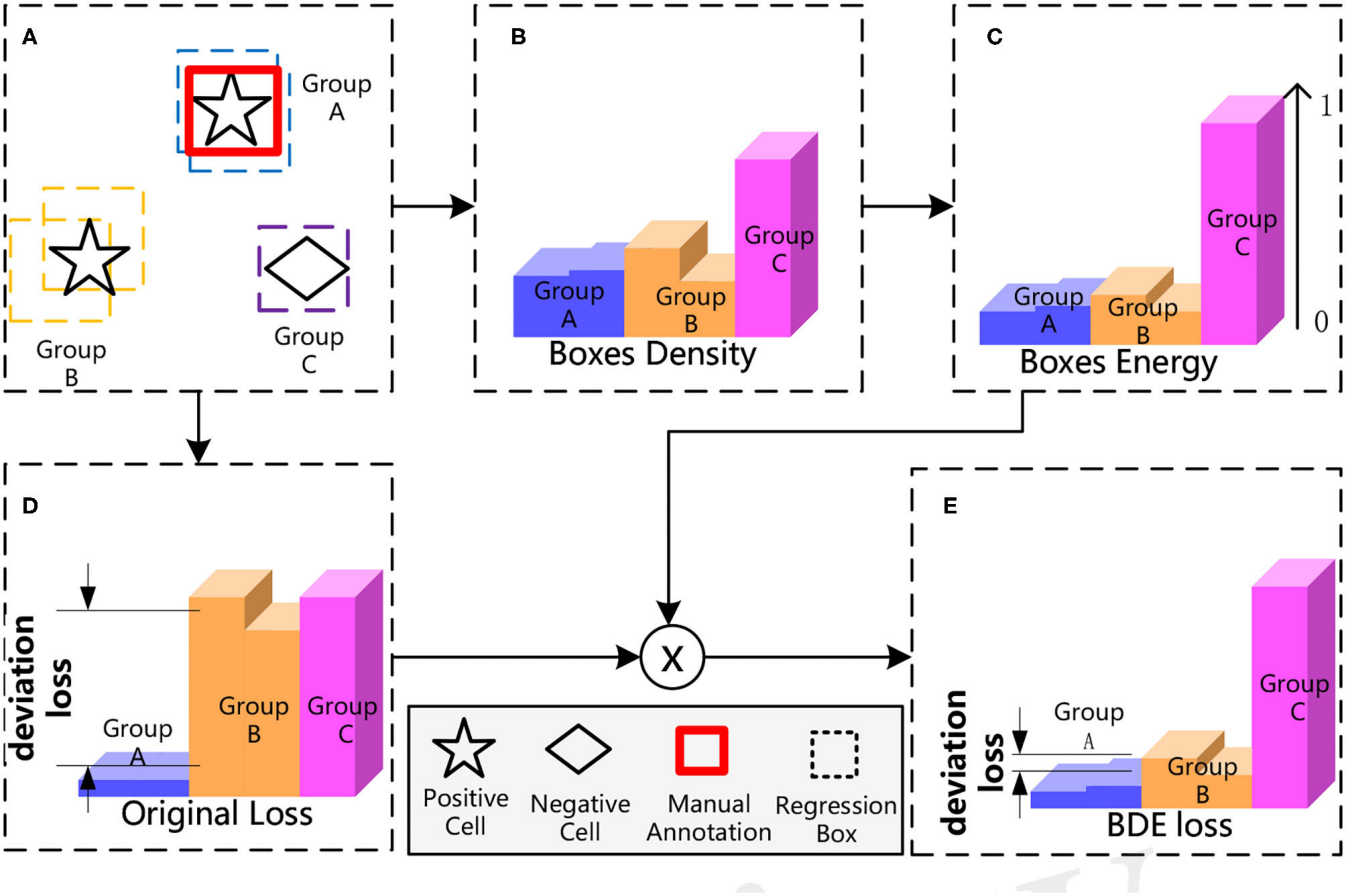
The process of Boxes Density Energy (BDE). The core idea of BDE is to use the distribution of regression boxes in **(A)**, to obtain the Boxes Energy of **(C)**, and to correct the deviation loss in **(D)**.

The overall process of our proposed BDE is shown in Figure 4. BDE is proposed to encourage the correct predictions of unannotated positive cells to ignore the adverse effect of the deviation-loss, which can be summarized into five core steps. Figure 4A A sparsely annotated image is inputted for the training. At the second stage of the detector, each cell is surrounded by some regression boxes automatically that we regard as a group. Figure 4B Boxes Density: Calculate the average distance between each box and the others. Figure 4C Boxes Energy: Normalized operation by dividing the Box Density by the maximum distance between all boxes. Figure 4D Calculate the original total loss. Figure 4E BDE loss: Calibrate the original loss with Boxes Energy to guide the detector training in the right direction.

### 3.1. Boxes Density

The boxes density can be measured by the average distance between each box, so that denser boxes have smaller average distances than isolating ones. The density of a box indexed by *i* can be represented as:

(5) $$\begin{array}{r} \mathrm{Density}\left(b_{i}\right)=\frac{1}{N}\overset{N}{\underset{j}{\sum }}D\left(b_{i},b_{j}\right), \end{array}$$

where *N* is the number of boxes per image, *D* is the distance function, we choose Manhattan distance (Equation 6) in this study considering the less computational cost.

(6) $$\begin{array}{r} D\left(b_{i},b_{j}\right)=|x_{i}-x_{j}|+|y_{i}-y_{j}|, \end{array}$$

In which, the *x*_*i*_ and *y*_*i*_ represent the x-coordinate and y-coordinate of the center point of the box indexed by *i*.

We can prove that the average distance can measure the density effectively; if we treat regression boxes around a cell as a group, and assume that we have *k* groups {*G*_1_, …, *G*_*j*_, …*G*_*k*_}. Meanwhile, there are {*m*_1_, …, *m*_*j*_, …, *m*_*k*_} boxes in the corresponding group.

For simplicity, we assume that the distances within a group are all close to 0, the distances between the groups are all *d*, and the total number of boxes is *N*, which means that $N=\overset{k}{\underset{l=1}{\sum }}m_{L}$. Thus, the average distance of each box in the *j*-group is Equation (7). This indicates that the box in a denser group (larger *m*_*j*_)of the *j*-group has a smaller density value.

(7) $$\begin{array}{l} \begin{array}{l} \mathrm{Density}\left(b_{i}\right)=\frac{0\times m_{j}+\left(N-m_{j}\right)\times d}{N}=d\times \left(1-\frac{m_{j}}{N}\right). \end{array} \end{array}$$

### 3.2. Boxes Energy and Loss Calibration

The main idea of our proposed method is that the more prediction boxes around a cell, the cell is more likely to be positive, and therefore, the predictions should have a smaller loss. The density of each box has been modeled, however, the range of density is not normalized. Therefore, we use Equation (8) to convert the Boxes Density to Boxes Energy which is normalized from 0 to 1. Afterward, Boxes Energy can be utilized as a weight of *L*_*cls*_ and *L*_loc_ (refer to Equations 9, 10). By that, the deviation loss is alleviated by calibrating the original loss.

(8) $$\begin{array}{r} \mathrm{Energy}\left(b_{i}\right)=\frac{\mathrm{Density}\left(b_{i}\right)}{\max\left(D\left(b\right)\right)}. \end{array}$$

(9) $$\begin{array}{r} L_{cls}^{BDE}\left(p,u\right)=\underset{k}{\sum }\left[1_{u_{k}=0}\left(Energy\left(b_{k}\right)\right)+1_{u_{k}\neq 0}\right]\cdot \left[-u_{k}\cdot \log\left(p_{k}\right)\right], \end{array}$$

(10) $$\begin{array}{l} \begin{array}{l} L_{loc}^{BDE}\left(b,v,u\right)=\underset{k}{\sum }\left[1_{u_{k}=0}\left(Energy\left(b_{k}\right)\right)+1_{u_{k}\neq 0}\right]\\ \;\cdot \left[{\mathrm{smooth}}_{L_{1}}\left(b_{k}-v_{k}\right)\right]. \end{array} \end{array}$$

In Equations 9, 10, *u*_*k*_ equals zero indicates the one-hot label of the box indexed by *k* is negative. With the loss-calibration of BDE, the detector can be trained along the right direction on the SADs. For example, if the box indexed by *k* is mistaken for negative (*u*_*k*_ is zero) due to SADs, but has a small *Energy*(*b*_*k*_), then, the original deviation-loss is calibrated by the term of *Energy*(*b*_*k*_). Finally, the total loss is improved from Equation (1) to:

(11) $$\begin{array}{r} L^{BDE}\left(p,u,b,v\right)=L_{cls}^{BDE}\left(p,u\right)+L_{\text{loc}}^{BDE}\left(b,v\right). \end{array}$$

## 4. Experiments

We utilize the FPN (19) with the backbone resnet50 (30) as the baseline. Our method is also compared with the representative loss-calibration methods, namely Label Smooth (LS) (27) and ProSelfLC (28). In Section 4.3, we conduct experiments to detect mitosis on the 2014 MITOS-ATYPIA Grand Challenge dataset and to detect tumor-cells on the Ki-67 dataset in Section 4.4. These two datasets can represent embedded and dense annotations. Experimental results demonstrate that BDE outperforms other methods on the SADs significantly, and BDE can address the training problem of SADs of both embedded and dense annotations.

### 4.1. Description and Implementation Details

The experiments for KI-67 and 2014 MITOS-ATYPIA datasets set the same hyperparameters. The inputted image is resized to the resolution of 800 × 800 pixels. The number of training steps is 10 k. The learning rate is initially set to 0.001 and is divided by 10 at 5 k and 7.5 k steps. In order to objectively evaluate our method, we perform 4-fold cross-validation on the MITOS-ATYPIA-14 dataset and 3-fold cross-validation on the Ki-67 dataset. We implement our framework with the open source software library TensorFlow version 1.12.0 on a workstation equipped with two NVIDIA GeForce 2080 Ti GPUs.

### 4.2. Evaluation Metrics

The average precision (AP) and recall are used for performance evaluation. The recall is defined as the proportion of all positive examples ranked above a given rank. Precision is the proportion of all examples above that rank that are from the positive class. The AP summarizes the shape of the precision/recall curve and is defined as the mean precision at a set of eleven equally spaced recall levels [0, 0.1,…, 1]:

(12) $$\begin{array}{r} AP=\frac{1}{11}\underset{r\in \left\{0,0.1,\ldots ,1\right\}}{\sum }p_{\text{interp}}\left(r\right). \end{array}$$

The precision at each recall level *r* is interpolated by taking the maximum precision measured for a method for which the corresponding recall exceeds *r*:

(13) $$\begin{array}{r} p_{\text{interp}}\left(r\right)=\underset{\tilde{r}:\tilde{r}\geq r}{\max}p\left(\tilde{r}\right), \end{array}$$

where $p\left(\tilde{r}\right)$ is the measured precision at recall $\tilde{r}$ (31).

### 4.3. Experiments on the 2014 MITOS-ATYPIA Grand Challenge Dataset (embedded annotations)

#### 4.3.1. Data Description

We have conducted experiments on the 2014 MITOS-ATYPIA Grand Challenge Dataset (MITOS-ATYPIA-14 dataset). The data samples were scanned by two slide scanners Aperio Scanscope XT and Hamamatsu Nanozoomer 2.0-HT, whole-slide histological images (WSIs) stained with standard hematoxylin and eosin (H&E) dyes. The centroids pixels of mitoses were manually annotated *via* two senior pathologists. In a situation of contradiction between the pathologists, the third one will provide the final say.

We choose the train-set of WSIs scanned from Hamamatsu Nanozoomer 2.0-HT, and we sample 393 patches that contain 743 mitoses with a sliding window of resolution of 1,663 × 1,485 pixels. Annotations for training the FPN are generated by 32 × 32 bounding boxes centered on all centroids pixels. For the MITOS-ATYPIA-14 dataset, we refer to the original data as a fully annotated dataset. Meanwhile, we randomly delete annotations until there is only one per training image and name it as an extremely sparse dataset. It is worth noting, we only conduct the sparse operations on the training dataset, and the testing dataset is intact.

#### 4.3.2. Results of MITOS-ATYPIA-14 dataset

Boxes Density Energy can improve recall results on the fully annotated dataset. Table 1 lists the recall and AP results on the fully annotated dataset. For the AP results, all methods have lower AP results than the baseline (FPN), which demonstrates that when loss-calibration methods are introduced to the training on fully annotated embedded annotations, interfering with the network's accuracy. On the other hand, for the recall results, BDE can improve the recall results significantly. FPN, LS, and ProSelfLC achieve 89.8, 85.5, and 88.7% average recall, respectively. While BDE achieves 94.6%, exceeding that of FPN by 4.8%.

**Table 1:** The recall and average precision (AP) results on the fully annotated MITOS dataset (original dataset).

**Method**	**Fold1**		**Fold2**		**Fold3**		**Fold4**		**Avg. Recall**	**Avg. AP**
	**Recall**	**AP**	**Recall**	**AP**	**Recall**	**AP**	**Recall**	**AP**		
FPN (Baseline)	80.2	41.8	89.4	46.9	95.8	44.6	93.6	60.7	89.8	**48.5**
LS (27)	75.6	36.7	84.6	47.7	91.6	41.7	90.4	64.2	85.5	47.6
ProSelfLC (28)	80.2	32.7	86.5	40.6	95.2	40.3	93.1	62.7	88.7	44.1
BDE (ours)	90.6	40.7	93.3	42.3	99.4	43.2	95.0	59.1	**94.6**	46.3

Boxes Density Energy improves the network's performance in all aspects on the sparsely annotated dataset. As shown in Table 2, BDE outperforms other methods significantly on both AP and recall results. However, LS's overall performance is reduced compared with the baseline, which indicates that the assumption of annotation-distribution of LS is incompatible in the embedded annotations, whose positive and negative samples are extremely unbalanced.

**Table 2:** The recall and AP results on the sparsely annotated MITOS dataset (retain one annotation in each image).

**Method**	**Fold1**		**Fold2**		**Fold3**		**Fold4**		**Avg. Recall**	**Avg. AP**
	**Recall**	**AP**	**Recall**	**AP**	**Recall**	**AP**	**Recall**	**AP**		
FPN (Baseline)	69.8	34.5	81.7	32.9	94.6	37.4	88.1	55.9	83.6	40.2
LS (27)	65.8	24.6	71.1	30.4	86.8	33.9	83.4	54.6	76.7	35.8
ProSelfLC (28)	80.2	28.8	84.6	28.4	95.8	30.1	85.7	50.1	86.5	34.3
BDE (ours)	88.5	41.8	89.4	37.1	95.8	40.2	91.3	60.1	**91.3**	**44.8**

### 4.4. Experiments on the Ki-67 Dataset (Dense Annotations)

#### 4.4.1. Data Description

The Ki-67 dataset is used for training FPN to detect tumor-cells and count their number. We have 206 patches with a resolution of 1,080 × 1,920 pixels sampled from WSIs, and the pathologists try their best to annotate all the tumor cells with key points in all patches. Finally, 21,025 tumor cells have been annotated. Then, we generate 32 × 32 bounding boxes centered on all key points.

##### 4.4.1.1. The SAD of the Ki-67 Dataset

For the Ki-67 dataset, considering that there is an average of 102 annotated tumor cells in each patch, so we can retain different annotation rates to train the network to fully validate BDE, e.g., the retentive rate is 0.1 if 10% of annotations are retained. We have carried out experiments starting from the retentive rate of 0.1 and increasing it to 1 by 0.1. We believe that if the retentive rate is below 0.5, then the dataset we can define as a SADs because the number of unannotated instances is greater than the number of annotated instances in such a dataset. Experimental results have demonstrated the BDE can significantly boost the performance of networks trained on that SADs.

#### 4.4.2. The Quantization Results

We evaluate the performance of our BDE which is trained on datasets with different retentive-rates, and observe that BDE is a robust training method, which is hardly affected by the quality of data annotations. For example, in Table 3, when the retentive-rate is dropped from 1.0 (original) to 0.1, BDE's AP result dropped from 49.02 to 46.45%, only reducing by 2.57%. On the other hand, FPN decreased by 23.88%, and LS decreased by 27.17%, and ProSelfLC decreased by 21.05%.

**Table 3:** The AP results on different annotations-retentive rates on the Ki-67 dataset.

**Retentive rate**	**0.1**	**0.2**	**0.3**	**0.4**	**0.5**	**0.6**	**0.7**	**0.8**	**0.9**	**1**
FPN (Baseline)	26.22	33.57	39.92	41.94	43.88	45.15	46.17	47.28	48.22	50.1
LS (27)	24.30,	37.39	41.01	44.16	45.48	46.47	47.69	48.96	50.01	51.47
ProSelfLC (28)	30.67	38.85	43.07	45.37	46.57	**47.72**	**48.79**	**49.91**	**50.87**	**51.72**
BDE (ours)	**46.45**	**46.36**	**46.24**	**46.71**	**46.94**	47.52	47.24	48.05	48.60	49.02

**Figure 5:**
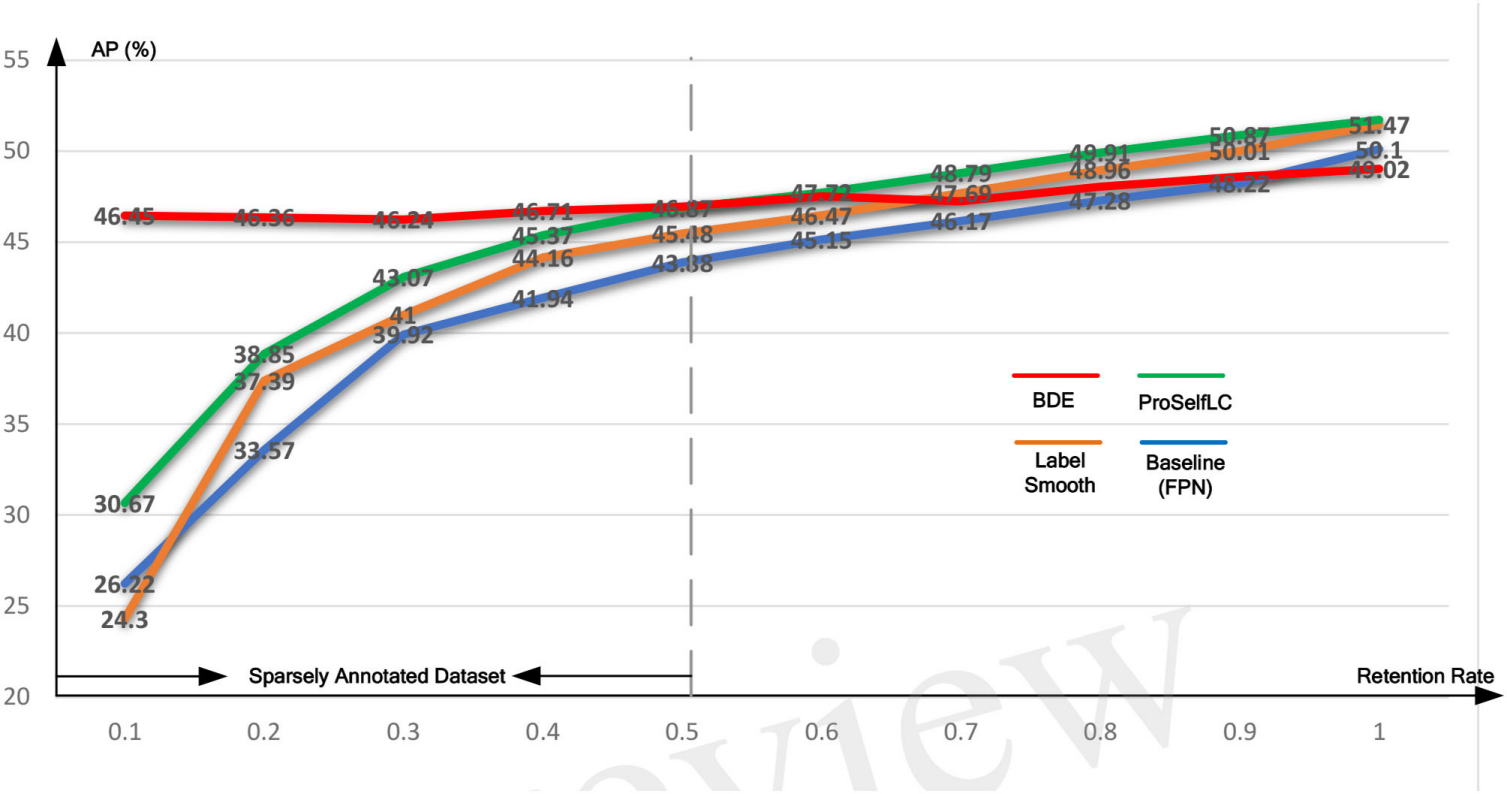
The average precision (AP) test-results curve of different methods trained on the Ki-67 dataset of different retentive rates. The horizontal coordinate stands for different retentive rates and the vertical coordinate for AP(%).

**Figure 6:**
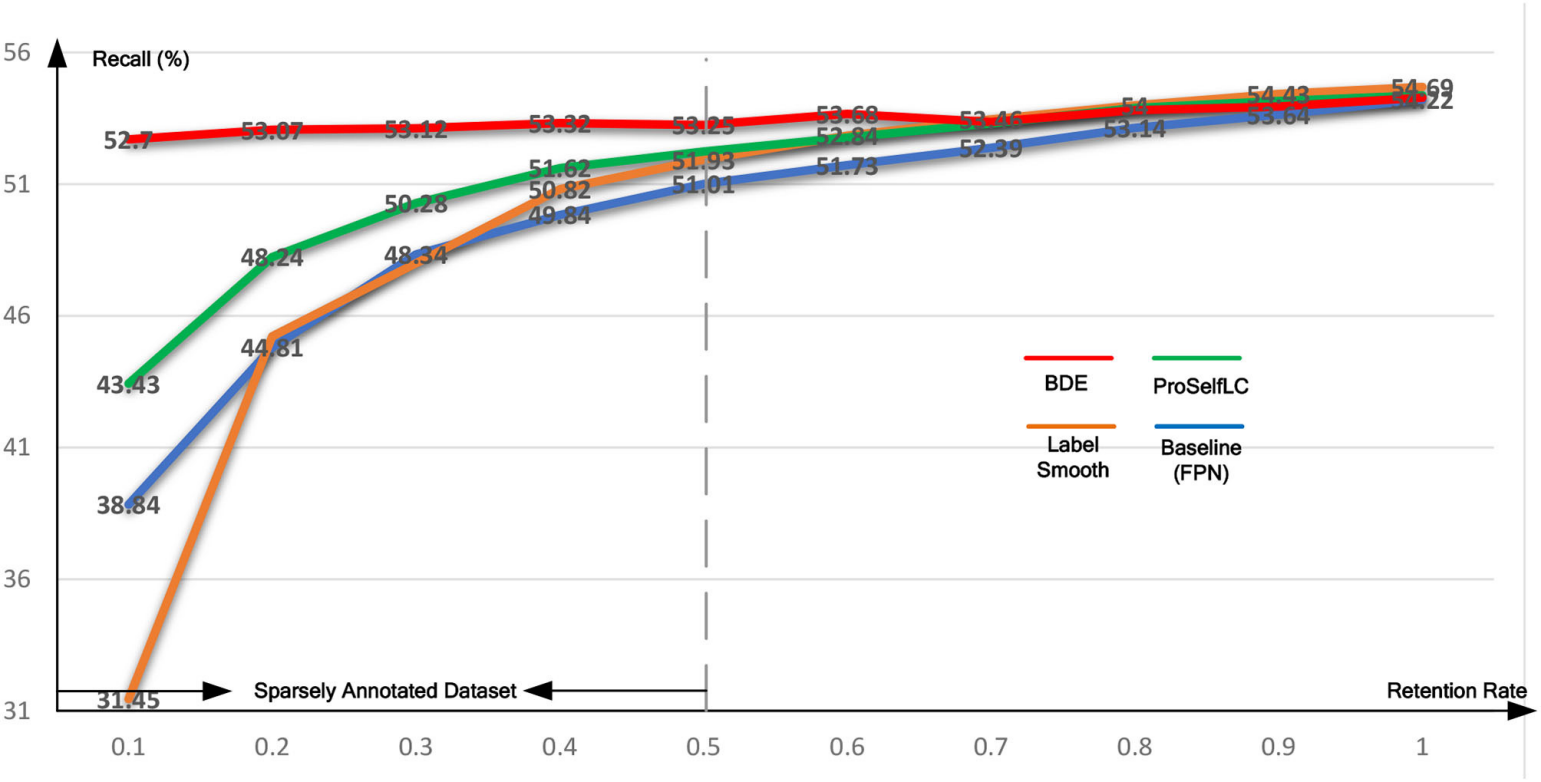
The recall test-results curve of different methods trained on the Ki-67 dataset of different retentive rates. The horizontal coordinate stands for different retentive rates and the vertical coordinate for recall(%).

Similarly, Table 4 lists the recall results of different methods trained on different retentive-rates. When the retentive-rate decreases from 1.0 to 0.1, BDE only reduces recall results by 0.59%. While FPN, LS, and Proself LC decreased by 15.38, 23.24, and 10.95%, respectively. Furthermore, from Figures 5, 6, the robustness and stability of BDE can be demonstrated from the perspective of AP results and recall results' curves. Our method is almost unaffected by sparse annotations. In particular, when the retentive rate is in the range of 0.1–0.5, that is, sparse annotation, BDE achieves significant improvements.

**Table 4:** The recall results on different annotations-retentive rates on the Ki-67 dataset.

**Retentive rate**	**0.1**	**0.2**	**0.3**	**0.4**	**0.5**	**0.6**	**0.7**	**0.8**	**0.9**	**1**
FPN (Baseline)	38.84	44.81	48.34	49.84	51.01	51.73	52.39	53.14	53.64	54.22
LS (27)	31.45	45.23	48.01	50.82	51.93	52.84	**53.46**	**54.00**	**54.43**	**54.69**
ProselfLC (28)	43.43	48.24	50.28	51.62	52.25	52.78	53.31	53.90	54.16	54.38
BDE (ours)	**52.70**	**53.07**	**53.12**	**53.32**	**53.25**	**53.68**	53.37	53.82	53.95	53.29

#### 4.4.3. The Qualitative Results

**Figure 7:**
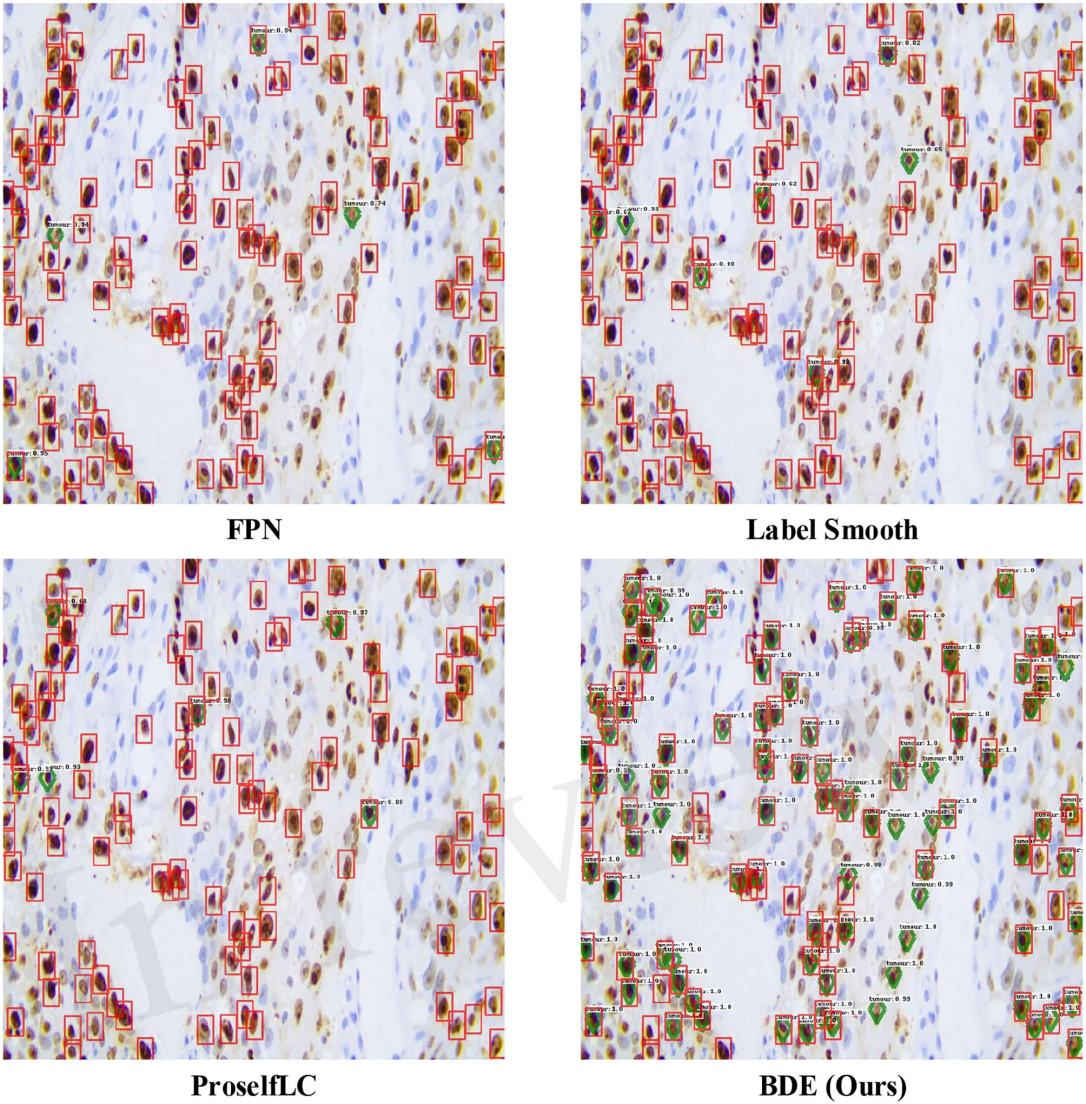
Examples of detection results on the Ki-67 dataset. These results are trained on the dataset with the retentive rate of 0.1 and tested on the test-set. Predictions are drawn in green diamond, and manual annotations are in red boxes. A score threshold of 0.6 is used for display.

In Figure 7, we list some detection results produced by different methods. A score threshold of 0.6 is used for display. Obviously, other methods trained on the sparsely annotated dataset (the retentive rates is 0.1) tend to miss tumor cells, while our method largely avoids that mistake. Meanwhile, our BDE trained on the 0.1 retentive rate even achieve better performance than other methods trained on the 0.4 retentive rate.

## 5. Layer-Level Gradient Analysis

### 5.1. Why Need Layer-Level Gradient Analysis

The gradient of a kernel is obtained by taking the chain derivative of the loss with respect to the weight, so that, the larger the weight of the kernel, not only its gradient is smaller but it also indicates that the kernel is more important. Thus, by comparing gradients of the same kernel but trained by different methods, we can know the advantages and disadvantages of training methods for this kernel. However, there are usually more than thousands of kernels in a single network, and it is not instructive to understand the superiority of kernel-level training. On the other hand, the same layer's kernels are responsible for similar feature extractions, e.g., kernels of a specific layer extract edges from different angles. Naturally, all kernels' average gradients in each layer can be used as an objective evaluation standard for feature extraction ability. Therefore, we analyze the gradient of each layer to investigate why BDE can improve the performance.

### 5.2. How to Analyze the Gradient

We analyze the mean value of the gradients in each layer of the network by computing the back-propagation *via* the testing loss. Specifically, for a layer indexed by *l*, whose mean gradient (μ_*l*_) can be computed as follow:

(14) $$\begin{array}{r} \mu_{l}=\overset{k=K}{\underset{k=1}{\sum }}\frac{1}{K}\cdot A_{l,k};\;\mu_{l}\in R^{1}, \end{array}$$

in which, *K* is the number of convolution kernels in the layer indexed by *l*, and *A*_*l,k*_ can be obtained by Equation (15).

(15) $$\begin{array}{r} A_{l,k}=\frac{1}{d\times w\times h}\overset{d}{\underset{i}{\sum }}\overset{w}{\underset{j}{\sum }}\overset{h}{\underset{m}{\sum }}G_{l,k}^{i,j,m}, \end{array}$$

where *G*_*l,k*_ is the gradient of k-th convolutional kernel in the l-th layer. Meanwhile, *d*, *w*, and *h* are the depth, the width, and the height of this kernel. *G*_*l,k*_ can be computed by Equation (16).

(16) $$\begin{array}{r} G_{l,k}=\overset{N}{\underset{i}{\sum }}\frac{1}{N}\cdot |\frac{\partial L_{test}^{i}}{\partial W_{l,k}}|;\;G_{l,k}\in R^{d\times w\times h}, \end{array}$$

where $L_{test}^{i}$ represents the loss computed on the *i*-th testing image, and there are *N* testing images, and *W*_*l,k*_ is the weights of the k-th convolutional kernel in the l-th layer. Further, the gradient represents the direction whether it is positive or negative, so that we perform an absolute operation on the calculated gradient.

### 5.3. Visualization and Discussion of the Gradient

**Figure 8:**
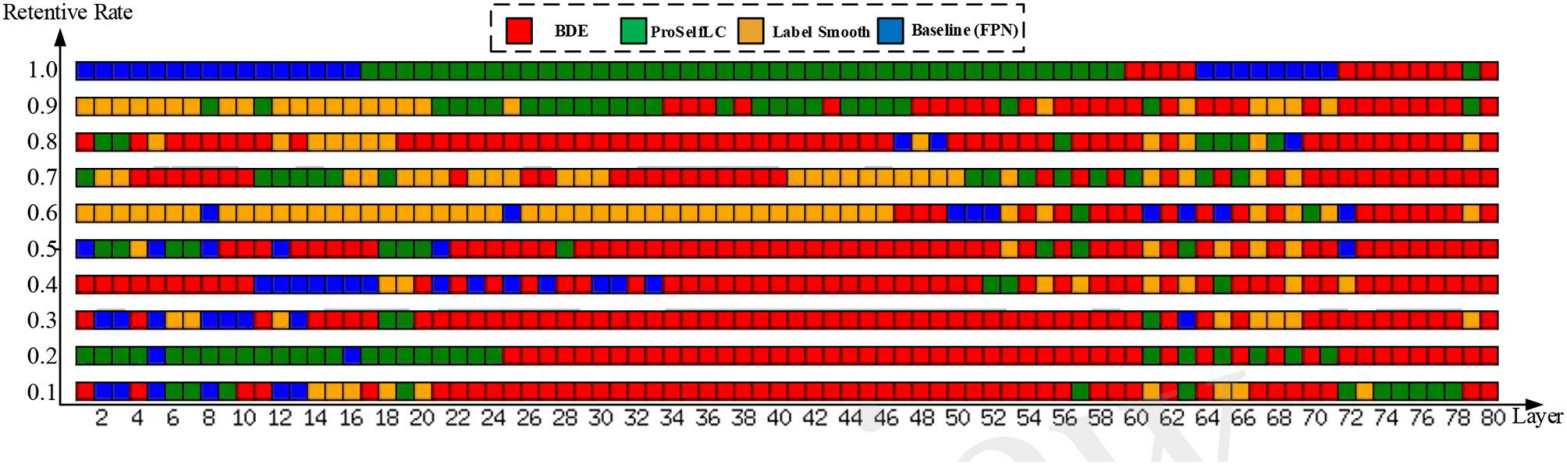
The gradient is visualized on the whole network (80 layers) with different training methods and different retention rate annotations (from 0.1 to 1.0, bottom to top). Each row in this figure contains 80 grids representing 80 layers, and the color of each grid encodes which training method obtains the minimum gradient at a layer, e.g., the red grid representing BDE has the minimum gradient for a specific layer.

As shown in Figure 8, we visualize the layer-level gradient of the networks (with 80 layers), which are trained on the Ki-67 dataset (retentive rates range from 0.1 to 1), and the gradient is obtained by the testing loss of the Ki-67 dataset. For each layer, we compare whose gradient is trained on different methods. Specifically, a grid with different colors indicates which method can obtain the minimum gradient, e.g., a red grid shows that our approach reduces the test gradient for a particular layer.

We can observe from Figure 8 when the network is trained on a dataset whose retentive rate below 0.5, BDE improves most of the middle layers (roughly 20–60 layers), which does not seem to happen by accident. Therefore, we can further presume that the generalization performance improvement of the cell detection task is closely related to the middle layers of the network.

## 6. Conclusion

In this study, through theoretical analysis and experimental verification, we identify that the detector trained on sparsely annotated cellular datasets may fall into overfitting due to deviation-loss. In order to address the training limitation, we propose a novel training method, which is utilized to calibrate the deviation-loss based on the cues provided by the density of regression boxes. Extensive experiments demonstrated the strength of BDE to significantly improve the training performance of the cellular detector, even with 90% of annotations missing, the performance of our method is barely affected. Thus, our proposed BDE might enable better and faster development of accurate cellular detection. More importantly, through the visual analysis of the network gradient, we find that the improvement of generalization performance is closely related to the middle layer of the network, which is expected to provide a new theoretical direction for future research.

## Data Availability Statement

The original contributions presented in the study are included in the article/supplementary material, further inquiries can be directed to the corresponding author.

## Author Contributions

HL: conception and design of study. FL, XS, and LH: drafting the manuscript. YK, ZW, and JL: analysis and/or interpretation of data. LY, WY, and QM: acquisition of data. LC and JF: funding acquisition. All authors contributed to the article and approved the submitted version.

## Funding

This study is supported by the National Natural Science Foundation of China (NSFC grant no. 6207326), and the Natural Science Foundation of Shaanxi Province of China (2021JQ-461, 2020JM-387).

## Conflict of Interest

WY, LH, and QM were employed by the company AstraZeneca, Shanghai, China. The remaining authors declare that the research was conducted in the absence of any commercial or financial relationships that could be construed as a potential conflict of interest.

## Publisher's Note

All claims expressed in this article are solely those of the authors and do not necessarily represent those of their affiliated organizations, or those of the publisher, the editors and the reviewers. Any product that may be evaluated in this article, or claim that may be made by its manufacturer, is not guaranteed or endorsed by the publisher.

## Figures and Tables

**Figure A1:**
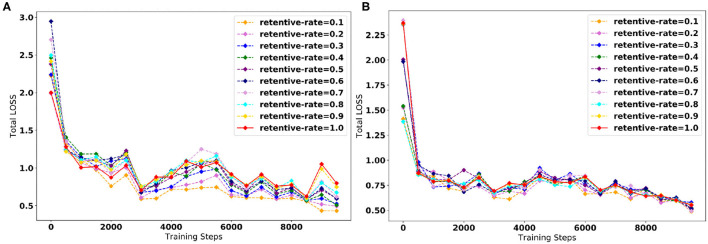
The loss curves for a standard detector and our method training under different cellular-level retentive annotation rates of the training dataset, smaller retentive rate indicates fewer annotations. **(A)** For a standard detector's loss curve. **(B)** The loss curve of our training method.

## Footnotes

^1^MITOS-ATYPIA-14 dataset: https://mitos-atypia-14.grand-challenge.org/dataset/.

## Appendix

### Overfitting Issues When Datasets Are Sparsely Annotated

Figure A1A in Appendix exhibits the loss curve of a standard object detector trained on the KI-67 dataset at different cellular-level retentive annotation rates. Before 3,000 steps, the detector trained on the datasets with a lower retentive annotation rate leads to a larger loss, which indicates that the deviation-loss dominates the training process. After that, lower retentive annotation rates lead to smaller losses, which indicates that the detector tends to focus on the annotated instances and then drives the overfitting issue. As shown in Figure A1B In Appendix, our method can significantly solve the overfitting issue.
